# The Empathic Brain of Psychopaths: From Social Science to Neuroscience in Empathy

**DOI:** 10.3389/fpsyg.2020.00695

**Published:** 2020-04-16

**Authors:** Josanne D. M. van Dongen

**Affiliations:** Department of Psychology, Education and Child Studies, Erasmus University Rotterdam, Rotterdam, Netherlands

**Keywords:** psychopathy, empathy, theory of mind, social neuroscience, complex brain networks, forensic mental health

## Abstract

Empathy is a crucial human ability, because of its importance to prosocial behavior, and for moral development. A deficit in empathic abilities, especially affective empathy, is thought to play an important role in psychopathic personality. Empathic abilities have traditionally been studied within the social and behavioral sciences using behavioral methods, but recent work in neuroscience has begun to elucidate the neural underpinnings of empathic processing in relation to psychopathy. In this review, current knowledge in the social neuroscience of empathy is discussed and a comprehensive view of the neuronal mechanisms that underlie empathy in psychopathic personality is provided. Furthermore, it will be argued that using classification based on overt behavior, we risk failing to identify important mechanisms involved in the psychopathology of psychopathy. In the last decade, there is a growing attention in combining knowledge from (neuro)biological research areas with psychology and psychiatry, to form a new basis for categorizing individuals. Recently, a converging framework has been put forward that applies such approach to antisocial individuals, including psychopathy. In this bio-cognitive approach, it is suggested to use information from different levels, to form latent categories on which individuals are grouped, that may better reflect underlying (neurobiological) dysfunctions. Subsequently, these newly defined latent categories may be more effective in guiding interventions and treatment. In conclusion, in my view, the future understanding of the social brain of psychopaths lies in studying the complex networks in the brain in combination with the use of other levels of information (e.g., genetics and cognition). Based on that, profiles of individuals can be formed that can be used to guide neurophysiological informed personalized treatment interventions that ultimately reduce violent transgressions in individuals with psychopathic traits.

## Introduction

Empathy is seen as the “natural capacity to share, understand, and respond with care to the affective states of others” (Decety, 2012). It plays an important role in social interactions, not only in humans, but also other species including apes (de Waal, 2012), and rodents (Decety et al., 2016). Moreover, empathy is thought to play an important role in affecting prosocial behavior, inhibiting aggressive behavior and is found to be fundamental to the development of moral behavior (Eisenberg and Eggum, 2009). Over centuries of literature on empathy has shown that empathy is sometimes confused with, or used interchangeably with other concepts, such as sympathy and compassion. In my view, empathy encompasses different facets and differs from sympathy and compassion in that empathy not only includes *other-oriented* empathy (i.e., empathic concern), but also entails *self-oriented* responses (i.e., emotional distress and emotional contagion). Thus, empathy differs from sympathy and compassion in the sense that it includes feelings that are similar *as* the other feels and not feelings *for* how the other person feels (Batson, 2009).

Since social sciences are concerned with different disciplines that examine society and how individuals interact with the social environment, empathy was originally studied within these disciplines. Psychology, the study of the human behavior and mind, has naturally focused on behavioral aspects of social interactions. For instance, behavioral research in social psychology has led to the empathy-altruism hypothesis (Batson, 2009). This hypothesis is supported on ample evidence that empathy is an other-oriented behavior, and is not egoistic in nature. Moreover, it is suggested that empathic concern for others results in altruistic motivation to care and help others.

Importantly, empathy is such an essential component of healthy human social interactions that absence of it may lead to severe social and cognitive dysfunctions. A personality structure often marked by a lack of empathy is psychopathic personality. Thus, clinical psychology is also concerned with the process of empathy and how this ability influences antisocial personality (including psychopathy) and behavior. And although manifestations of personality and psychopathology in psychology is originally studied from a behavioral point of view (i.e., symptoms), psychological science is integrating the neurobiological underpinnings of cognition and behavior. Also, in the study of psychopathic personality, scholars become more aware of the fact that psychopathic personality is heterogeneous, consisting of multiple facets of traits with each of these traits having different underlying neuro-cognitive processes.

Alternative approaches to study personality and psychopathology have emerged decades ago (see for example Morton and Frith, 1995 on causal modeling). Also, approaches that incorporate neuroscience, such as the Research Domain Criteria (RDoC; Insel et al., 2010), have emerged already in the nineteenth century (Arzy and Danziger, 2014). However, these approaches have not been applied to the study of psychopathic personality more specifically, only until a couple of years ago (Blair, 2015a,b; Brazil et al., 2018). The idea behind these approaches is that mental disorders are originally classified based on behavioral symptoms (e.g., DSM criteria), but that, during the last decades, it has become increasingly apparent that these disorders consist of dysfunctional bio-cognitive processes related to different processes at the neural level. Each of these processes are found to be existent transdiagnostically, and therefore must be studied individually. In the case of empathy, this is not only dysfunctional in psychopathic personality, but also in autism, schizophrenia, and borderline personality disorder (Lockwood, 2016).

Thus, elucidation of the neural underpinnings of empathy will not only help us understand social interactions, but also help us understand the neural and cognitive mechanisms of emotion processing, motivation (i.e., empathic concern), and individual differences in antisocial and psychopathic personality.

The aim of this review paper is to give an overview of our current knowledge on the role of neuroscience in the study of empathy in psychopathic personality. First, some conceptual matters of empathy and associated concepts are clarified, and it is argued that the construct of empathy needs to be defined by several subcomponents and processes that are underpinned by diverse processes in the brain. Additionally, studies on the neural circuits involved in empathy are reviewed. Next, a short historical overview of psychopathy as a construct will be given, as well as different theoretical models on this personality. In the third section, a review of empirical evidence is given that supports the role of social neuroscience in psychopathic personality. Finally, I will discuss a new way forward in using neuroscience in the study of the “empathic brain” of psychopaths.

## Empathy

As already mentioned in the introduction, the term “empathy” is applied to various phenomena, including feeling the same as another person is feeling, feeling pity for another person, and knowing what the other person is feeling or thinking (Batson, 2009). The labels of these concepts also vary between empathy, sympathy, pity, and compassion. Although these concepts are related, and sometimes overlap, they do not represent the same psychological (and neurobiological) phenomena. Not surprisingly, there is still a debate on what the construct of “empathy” entails. Some scholars include both self- and other-oriented processes (Decety, 2010), and others only include those phenomena that are oriented toward the person in need (other-oriented; empathic concern; Batson, 2009).

Hence, as already briefly outlined above, empathy (the capacity to understand and know the difference between one’s own emotions and feelings and that of another person) is distinguished from sympathy (to be concerned about the wellbeing of another person). While the terms empathy and sympathy are often used interchangeably, the two can be differentiated: the experience of empathy can lead to different outcomes: an *other-oriented* motivation, sympathy, or a *self-oriented* feeling of distress imposed by the stressor which includes, and may also be congruent to the emotional state of that other person (emotional contagion). Sympathy may be the result of understanding another’s affective state but does not have to be consistent with that state. Given the complexity of the experience of empathy, it is important to first break down this construct into component processes.

### The Components of Empathy

Generally, researchers have postulated that empathy includes both affective and cognitive components (Decety and Jackson, 2004; Eisenberg and Eggum, 2009; Decety, 2010; Zaki and Ochsner, 2012). Based on evidence from cognitive neuroscience and developmental psychology, a number of different, but interacting mechanisms result in the experience of empathy (Decety, 2010; Zaki and Ochsner, 2012): (1) An affective component of affective sharing or emotional contagion; a bottom-up process which is a result of perception-action coupling, and emotion perception (Preston and de Waal, 2002). (2) A cognitive aspect of mentalizing or perspective taking (i.e., Theory of Mind; ToM); the ability to make a distinction between oneself- and other, and (3) executive functions which influence the extent of an empathic experience, and results in empathic concern (i.e., sympathy), using amongst others the perceiver’s motivation, memories, and intentions.

Research indicates that the affective empathy develops before cognitive empathy. Following the Perception-Action Model (Preston and de Waal, 2002), it is suggested that newborns are able to mimic facial expressions, and infants are found to become distressed if they hear another baby cry. That is, they perceive the crying of another infant that (automatically) contributes to affective sharing. Thus, affective responsiveness is present at an early age, is automatic, and is the result of mimicry and somato-sensorimotor resonance between the self and other.

The cognitive components of empathy include ToM, or mentalizing. This is the ability to infer the mental states of another person, which includes executive functions such as attention, working memory, and self-regulation. These “higher” cognitive abilities are suggested to develop later in life, because the prefrontal cortex develops more slowly than more basal (emotion related) brain areas, reaching maturation in late adolescence (Bunge et al., 2002). The development of the prefrontal cortex permits children to express their feelings and develop self-regulation by using inhibitory control over their thoughts, attention, and actions (Diamond, 2002). Thus, although affective aspects of empathy develop early in life, maturation of the frontal brain influences the way executive functions interact with empathic responding. That is, executive functions (i.e., emotion regulation, inhibitory control, etc.) have their effect on how empathy develops in its full scope of facets.

Although at first it was thought that ToM abilities develop later in childhood, more recent studies have suggested that babies already have obtained these abilities to some extent by the age of 4 years (Onishi and Baillargeon, 2005). Moreover, babies as young as 7 months are found to have a “social sense” (Kovács et al., 2010). This social sense is an automatically computed *online* belief about another agent, which is maintained even in the absence of that agent.

### Sharing Emotions With Others

The perception and resonance of the affective states of another person are thought to result in shared representations of oneself and others. Evidence suggests that for particular emotions, such as fear, disgust and pain, there are brain regions that map the emotions of another to oneself. That is, we not only “simply” understand the emotions of another person, we also *feel as* and *feel with* the other person. These abilities are found to be grounded in shared representations (Keysers and Gazzola, 2006). However, although the human mind has, in some cases, an egocentric bias (we think that others think and feel as we think and feel), successful social interactions partly result from the ability to distinguish oneself from the other (Sommerville and Decety, 2006).

The shared-representation theory of social cognition (Sommerville and Decety, 2006) suggests that the experience of emotion in oneself and the perception of another’s emotions draw on many of the same underlying neural circuits and computational processes, including somatosensory and motor representation (see later in this review for neural structures and mechanisms involved in empathy). As will be discussed later, one important mechanism involved in this shared representation, is the mirror neuron system (Rizzolatti and Craighero, 2004; Iacoboni et al., 2005).

Past research generally has focused on “what is shared” by these shared representations (i.e., cognition and/or emotional states), and less on “how these are shared.” Advances have been made by Bird and Viding (2014), who formulated a model of mechanisms by which the affective state in another may result in an empathic response in the self. In this Self Other Model of Empathy (SOME), empathy is differentiated from emotional contagion in that emotional contagion results from the vicarious experience of the affective state of another person, *without* recognizing this state as being a part from that other person. Empathy results from the mechanisms of emotional contagion, with the addition that one recognizes that the experienced affective state is experience by that other person. This accomplished by a so-called Self/Other switch, a system that requires information from the ToM system to results in a switch from self (the default) to the other (Bird and Viding, 2014). Together with understanding the situation both the self and the other are in, it evaluates whether the affective state of the self, corresponds to the situation and emotional state of the other person.

### Neural Circuits in Empathy

Neuroscientists have started to elucidate the neurobiological underpinnings of empathy (Decety, 2010; Zaki and Ochsner, 2012). Functional neuroimaging studies have shown that imagining emotional experiences from our own and from someone else’s perspective result in comparable psychophysiological reactions and patterns of brain activation. For example, Ruby and Decety (2004) presented participants with short written scenario’s depicting real-life situations (e.g., someone opens the toilet door that you have forgotten to lock) which induce social emotions (e.g., shame, guilt, pride), as well as emotionally neutral situations. Subsequently, they asked them to imagine how they would feel if they were in those situations, and how their mother would feel in those situations. Results showed that the imagined emotional conditions for both the self and the other perspectives led to similar activation of brain areas that are involved in emotional processing, including the amygdala and the temporal poles.

In a study by Preston et al. (2007), heart rate, skin conductance, and neuroimaging measurements were combined in participants who were also asked to imagine a personal experience of fear or anger from their own past, and an equivalent experience from another person as if it were happening to them. Results confirmed earlier results, in that similar patterns of psychophysiological and neurological activation were found when participants could relate to the scenario of the other, and to those of personal emotional imagery.

Developmentally, the process of empathic distress or emotional distress may play a role in the underpinnings of prosocial behavior (Hoffman, 1990). Also, the expression of pain offers an important signal to others, that motivates behavior such as caring for a person in distress (i.e., sympathy). It is the affective experience of pain that indicates an aversive state and motivates behavior that, for example ends, or reduces exposure to the source that has led to the aversive state in the first place (Price, 2000). The perception and experience of pain is therefore often used by researchers as a valuable and ecologically valid means to investigate the experience of empathy.

Following the above, most research in empathy has focused on empathy for pain, and how different factors modulate its experience and behavioral expressions (Singer and Lamm, 2009; Lamm et al., 2011). For instance, as was already indicated in the paragraph above, different functional neuroimaging studies have shown that similar brain regions are activated during the personal experience of pain and when attending to the pain of others (Lamm and Majdandžiæ, 2015; Zaki et al., 2016). These regions include the anterior insula (AIC), anterior mid and dorsal anterior cingulate cortex (ACC), and periaqueductal gray (Lamm et al., 2011). In one functional magnetic resonance imagining (fMRI) experiment, participants were scanned during a condition of feeling a moderately painful pinprick stimulus to the fingertips and another condition in which they watched another person’s hand undergo similar stimulation (Morrison et al., 2004). Both conditions resulted in increased activity in the right dorsal ACC. Another fMRI study with healthy participants showed that the dorsal ACC, the AIC, cerebellum, and brain stem were activated both when the participants experienced a painful stimulus, as well as when they observed the same in another person receiving it. However, only the actual experience of pain resulted in activation in the somatosensory cortex and a more ventral region of the ACC (Singer et al., 2004). Additionally, these results are supported by two other fMRI studies (Jackson et al., 2005, 2006).

In a study by Zaki et al. (2007), participants were scanned while they received hurtful thermal stimulation (self-pain condition) or watched short videos of other people receiving painful stimulation (other pain condition). With connectivity analyses, the researchers found areas whose activity covaried with ACC and AI activity during self or other pain either across time (intra-individual connectivity) or across participants (inter-individual connectivity). Both connectivity analyses revealed clusters in the midbrain and periaqueductal gray with greater connectivity to the AI during self-pain as compared to other pain. Greater connectivity to the ACC and AI during other pain than during self-pain was found in the dorsal medial prefrontal cortex, using both types of analysis. Intra-individual connectivity analyses also revealed regions in the superior temporal sulcus, posterior cingulate, and precuneus that became more connected to ACC during other pain compared with self-pain. These and other results show that there are distinct neural networks associated with ACC and AI in response to personal experience of pain and response to seeing other people in pain (Morrison and Downing, 2007; Zaki et al., 2007).

Facial expressions of pain form an important category of facial expression that is easily comprehended by observers. In one study Botvinick et al. (2005), the neural response to these facial pain expressions were examined using fMRI while subjects viewed short video sequences showing faces expressing either moderate pain or, for comparison, no pain. Facial expressions of pain were found to lead to cortical activation similar to areas activated in firsthand experience of pain, including the ACC and AI. Similar results were found by Lamm et al. (2007), who scanned participants, and let them listen to painful sounds and let them watch videos of people expressing pain due to listening to painful sounds.

Concerning the brain structures involved in empathic experiences, the mirror-neuron system (MNS) and somatosensory cortex are suggested to be involved in experiencing and seeing the actual cause of pain (Decety, 2010). However, it remains debated whether the emotion sharing mechanism in humans actually requires the involvement of the MNS (Baird et al., 2011). Mirror neurons are a class of cells that were first identified in monkeys (Gallese et al., 1996). Although first it was thought that these cells were mainly involved in action understanding and imitation, now, different higher cognitive functions have been found to be associated to the MNS, including empathy (Rizzolatti and Craighero, 2004).

On the contrary, however, a conceptual analysis by Jacob (2008) of empirical research on mirror neurons and their assumed contribution to empathy, concluded that motor resonance (as a result of MNS activity), is neither necessary nor sufficient for representing another individual’s intentions. It was argued that mirror neurons may be best interpreted as motor system facilitators (Hickok, 2009). Their involvement in empathy may then be via the so-called “mimicry” (Decety, 2010) that is suggested to be necessary for perception-action coupling (Preston and de Waal, 2002). Subsequently, the ACC and AIC are associated with the affective value of somatosensory stimuli within this emotion sharing network (Singer et al., 2004; Keysers et al., 2010).

In sum, previous functional neuroimaging studies indicate that perceiving or imagining another individual in pain is associated with activity in brain areas processing sensory, and motivational-affective dimensions of pain in oneself.

## Psychopathy: an Overview

Psychopathy is a personality consisting of characteristics including callousness, lack of guilt, shallow affect, impulsive and antisocial behavior (Cleckley, 1976). Approximately 1% of the general population, but 20–30% of the prison population are found to have a psychopathic personality (Hare, 1999). Because of their behavioral characteristics, psychopathic individuals pose great costs to society (i.e., economically, mental healthcare, and criminal justice), estimated at $400 billion in the USA alone (Kiehl and Buckholtz, 2010). This seems to be comparable within European countries, such as the Netherlands, where treatment costs of antisocial offenders in forensic psychiatric facilities is $160,000 a year per person. These costs are extremely high, especially when compared to costs related to other diseases, such as treating type 2 diabetes, which is estimated at only $1,700–2,100 a year per person (Brandle et al., 2003).

Because of the high costs, both financially, but also emotionally, that psychopathic individuals pose, there is a strong need for classifying these individuals and developing treatment interventions that will target this personality. Unfortunately, as reflected by their high risk of recidivism, psychopathic individuals account for the majority of failed treatment efforts. Several attempts have been made to treat antisocial individuals, including those with psychopathic personality, using a variety of clinical approaches (Harris and Rice, 2006; Gibbon et al., 2010; Salekin et al., 2010). While there is some support for successfully targeting some characteristics of this personality using psychological and pharmacological treatment, there is no evidence that current treatments effectively address this personality. Therefore, some clinicians and researchers have postulated that individuals with elevated levels of psychopathy, maybe even untreatable (Harris and Rice, 2006). However, I think that the development of effective treatment interventions may be advanced by recognizing the heterogeneity of psychopathic personality and incorporating knowledge about the underlying neurobiological correlates of this personality into the development of more specific treatments.

### Subtypes of Psychopathy

Cleckley’s (1976) *The Mask of Sanity* served as a groundwork for different conceptualizations and measurements of psychopathic personality. Hare (1991, 2003) used Cleckley’s description of clinical criteria as a basis for the development of a diagnostic instrument for the assessment of psychopathic personality. The Revised version of Hare’s Psychopathy Checklist (PCL-R), an interview and file-based assessment instrument, is still regarded as the “golden standard” for assessing psychopathy in forensic and correctional settings. Generally, a score of 30 or above out of 40 (maximum score), is regarded as a cutoff for the classification as a psychopath. In European countries however, a cutoff score of 25 is being used. The PCL-R measures psychopathy in terms of two broad factors: Factor 1, including Affective and Interpersonal facets (i.e., grandiosity, deceitfulness, lack of empathy, and lack of remorse) of psychopathy, and Factor 2, including Antisocial and Lifestyle facets (i.e., deficit in behavioral inhibition and control).

Throughout the years, a lot of research has been conducted on the usefulness of the PCL-R and its different variants (Neumann et al., 2007). Like any assessment instrument, it has certain limitations. One is that several of its items refer directly to criminal activity, which makes the PCL-R less appropriate for use in non-correctional samples. Another is that the PCL-R is very time consuming to administer, and impractical for large scale data collection efforts because of its interview-based procedure and requirement of collateral (i.e., archival file) information. As a result, different other (self-report) measures are developed for the assessment of psychopathic personality during the years, some of them found to be more promising than others.

The term psychopathy has commonly been used as a unitary construct and to refer to one particular group of individuals scoring higher than a cut-off score on the PCL-R (Hare, 2003). The problem with assuming psychopathy as a unitary personality construct, is that it does not consider that persons scoring high and low on particular characteristics of psychopathy such as impulsivity, empathy and even anxiety are different from one another (Skeem et al., 2003). Nowadays, many researchers view psychopathic personality as being multidimensional, and believe that this personality includes multiple subtypes that differ significantly in etiology and personality characteristics (e.g., Skeem et al., 2003; Patrick et al., 2009).

During the last decades, different self-report measures of psychopathy are developed, to overcome some of the (practical) difficulties that come with the use of the PCL-R. These include for example the Self-Report Psychopathy Scale and its Short Form (SRP; Hare, 1980; SRP-SF; Paulhus et al., 2016), the Psychopathic Personality Inventory and its revised version (i.e., PPI; Lilienfeld and Andrews, 1996; and PPI-R; Lilienfeld and Widows, 2005), and the Levenson Self-Report Psychopathy Scale (LSRP; Levenson et al., 1995). One of the alternative frameworks of psychopathy that addresses the above multiple psychopathy types principle, is the Triarchic Psychopathy Model. Patrick et al. (2009) have proposed this conceptualization based on the observation that previous literature reveals three important facets within the construct of psychopathy: *boldness* (reduced emotionality, resilience to stress, and social dominance), *meanness* (lack of empathy, cruelty, and aggressive behavior toward others), and *disinhibition* (impulsivity and dysregulation of negative affect) (but see Roy et al., 2020 for a septarchic structure of this model). These three constructs are viewed as connected, yet distinct from one another, and can be measured and understood separately. The assumption is that the three dimensions can be combined to create descriptions for different subtypes of psychopathic personality. This approach also claims to account for adaptive features seen in psychopathy (i.e., boldness), traits that were incorporated in classic accounts of psychopathy (Cleckley, 1976; Lykken, 1995), which are not incorporated in the PCL–R. The construct of meanness, but also boldness to some extent, has theoretical relations with the concept of empathy. While meanness is viewed as the core construct associated with a lack of affective empathy (Sellbom and Phillips, 2013; Stanley et al., 2013), the concept of boldness does also entail fearlessness and the ability to remain calm in the face of threat, suggesting a negative relation to the personal distress facet of empathy. However, for an individual to show these boldness traits, this individual also needs to have (high) functioning mentalizing ability to successfully manipulate others.

## Social Neuroscience of Empathy in Psychopathy

### Theoretical Accounts

As described in previous paragraphs, individuals scoring high on psychopathic traits are defined as fearless, callous and have a lack of empathic disregard for others combined with impulsive and antisocial behavior (Hare, 2003). Also, it is found that they have difficulty controlling their emotions and often lack fear when facing punishment. Insights into neural circuits underpinning healthy empathic behavioral processes may shed light on potential neural dysfunctions in psychopathic personality. Conversely, advances made in the description of the component processes underlying psychopathic personality are invaluable as a complement to other methods of empathy research.

Different accounts have been formulated that explain psychopathic personality and its consecutive behavior. On the one hand are accounts that explain psychopathic personality on the basis of deficits in emotions, most notably anxiety and fear. In these theories it is argued that psychopathic individuals lack fear responses when faced with stressful situations and therefore do not form punishment related associations (Fowles, 1988; Patrick et al., 1994; Lykken, 1995). These theories are based on research that has shown deficits in emotion recognition (Marsh and Blair, 2008; Dawel et al., 2012), and (neuro)physiological responses to fear (Patrick et al., 1994; Kiehl et al., 2001).

On the other hand are accounts that are based on attentional deficits (i.e., the Response Modulation Hypothesis; Newman et al., 1987; Newman, 1998). In these theories, it is argued that deficits in psychopathic personality relate to difficulties in reallocating attention to information that is not relevant when engaged in goal-directed behavior. These attention views are partly based on findings that have shown that fear deficits seen in psychopathy are moderated by attention (Newman et al., 2010; Baskin-Sommers et al., 2011).

The Integrated Emotion Systems (IES) model (Blair, 2007, 2013), follows work that has been done within the emotion deficits approach, such as work from Patrick et al. (1994). This model stresses the importance of the amygdala. Research has shown that the amygdala is critical for stimulus-reinforcement learning, for example in aversive conditioning, which is impaired in psychopathy (Rothemund et al., 2012). This finding corresponds to findings that have shown that psychopaths show reduced activation of the amygdala during aversive conditioning (i.e., Birbaumer et al., 2005). In addition, the IES model also stresses the importance of the ventro-medial prefrontal cortex (vmPFC) including the orbitofrontal cortex (OFC).

Following this, according to the IES model, processing of emotional stimuli is involved in (moral) behavioral transgressions. Transgressions are learned to be considered as “bad” because of the aversive feedback that follows that transgression, for example the distress of the victims of these transgressions. Impaired stimulus-reinforcement learning as the result from amygdala dysfunction, and impaired responsiveness to the distress of others (e.g., communicated by facial expressions; Blair, 2011) lead to deficits in empathy for others and subsequently to (moral) behavioral transgression.

In support of the IES model, the amygdala is found to be important for processing expressions of fear and distress (Murphy et al., 2003), and individuals with psychopathy who are violent show reduced amygdala responses to fearful expressions (Dolan and Fullam, 2009). This dysfunctional response reflects a dysfunction in empathic responding (i.e., personal distress). Consequently, dysfunction in stimulus-reinforcement learning, thus learning the consequences (fear expression) of one’s actions (aggression), results in a deficient response to transgressions (i.e., empathic concern). Different studies found reduced amygdala responses follow moral transgressions and moral decision-making in individuals with psychopathic traits (Glenn et al., 2009; Harenski et al., 2010).

In line with the IES model, the violence inhibition model (VIM; Blair, 1995, 2001) also views empathy as an important mechanism for moral socialization. The VIM in addition accounts for the inhibition of violent behavior (or the lack of inhibition of that behavior) by coupling the activation of the mechanism by distress cues with representations of the acts which caused the distress cues (i.e., transgressions). A child that is developing appropriately thus initially finds the pain of others’ aversive and then, through aversive conditioning (or stimulus reinforcement), transgressions are inhibited because of the aversive consequences of that action. According to the VIM, individuals with psychopathic personality have dysfunctional neural circuits (i.e., the amygdala and vmPFC) involved in these associative learning mechanisms (Blair, 2001).

In support of the above, Greene et al. (2001) found that personal as opposed to impersonal moral choices let to increased vmPFC activity. Likewise, Luo et al. (2006) showed that in response to more severe moral transgressions, amygdala and vmPFC activity was increased when compared to less severe moral transgressions.

Following the IES model and the VIM, Blair (2007, 2008) argues that, while the amygdala is particularly involved with emotional responding and forming the learning basis of necessary for caring for the welfare of others, the vmPFC is particularly involved with the decision process following input from the amygdala. This corroborates with the idea that affective empathy (i.e., affective arousal/personal distress) is found to be mediated by subcortical structures from the limbic system, such as the amygdala. And, emotional decision-making, and subsequently empathic concern for others (including moral cognitions), are found to be mediated by the vmPFC (Decety, 2010).

### Functional Neuroimaging Studies

Neuroimaging studies found that above mentioned structures relevant for empathy are dysfunctional in persons with psychopathic traits (e.g., Koenigs et al., 2007; Shamay-Tsoory et al., 2010; Decety et al., 2013b; and see Lockwood, 2016 for a review). For instance, in one study, persons scoring high and low on the PCL-R were examined during the viewing of pictured depicting bodily harm (Decety et al., 2013a). They had to imagine that this harm involved oneself, or another person. During the imagine-self perspective, participants with higher scores on psychopathy showed atypical response in the AI, aMCC, SMA, IFG, somatosensory cortex, and right amygdala. This corresponds with the brain network involved in the experiencing of pain. Conversely, during the imagine-other perspective, individuals with higher scores on psychopathy showed a different pattern of cortical activation and effective connectivity resulting from the AI and amygdala with the OFC and vmPFC. Moreover, the imaging-other condition, response in the amygdala and insula was inversely correlated with the interpersonal and affective traits of psychopathy.

Meffert et al. (2013) conducted a study using fMRI involving the viewing of scenarios depicting hand movements and found a similar pattern of reduced activation of brain areas involved in empathy in persons with psychopathy compared with controls. Interestingly however, they also found that when these individuals were instructed to empathize with the person in the videos, the reduction in activation became less. The authors concluded that persons with psychopathy do not have a total absence or incapacity to empathize with another person, but that brain mechanisms involved are not automatically activated in these individuals (see also Keysers and Gazzola, 2014 on the ability vs. propensity for empathy). That persons with psychopathic traits do not seem to have a total lack of empathy was also shown by a recent online survey study (Kajonius and Björkman, 2020). In this study, the authors investigated the disposition of empathy and the ability to empathize in persons scoring higher and lower on the Dark Triad personalities (i.e., Machiavellianism, psychopathy, and narcissism). It was found that dark triad personality was not related to ability-based empathy, but strongly negatively related to dispositional based empathy.

With respect to the different facets that make up empathy and psychopathy, it may be of importance that most research that support a lack of empathy in psychopathy are supporting a lack of affective empathy. Robinson and Rogers (2015) for example, found that psychopathic criminals had no impairment in cognitive empathy (i.e., ToM or mentalizing), but did not seem to possess affective empathy. Likewise, Sandoval et al. (2000) found a negative relationship between self-reported affective empathy and psychopathy, but no relationship with cognitive empathy. However, there are also studies in which no relations or negative associations were found between both affective and cognitive empathy and psychopathy (Brook et al., 2013; Brook and Kosson, 2013; Domes et al., 2013).

Though ToM has been regarded as a cognitive aspect of empathy, according to the theoretical framework of Shamay-Tsoory et al. (2010), ToM is a construct that can be separated into cognitive and affective aspects. Cognitive ToM resembles what is generally referred to as metalizing, while the affective part refers to the ability to infer on other’s feelings and therefore relates to both affective and cognitive empathy. It is important to note that affective ToM differs from affective empathy, in that affective empathy also includes emotional contagion (feeling the same feeling as the other person does), while affective ToM does not.

Thus, when interpreting previous findings concerning the relation between psychopathy and empathy (including ToM), it is important to recognize the above mentioned difference in cognitive and affective ToM. As previously stated, most research found no lack of cognitive empathy in psychopathic individuals (Blair, 1996; Richell et al., 2003; Dolan and Fullam, 2009), while Brook and Kosson (2013) did find a lack of ToM in psychopaths. However, this lack of ToM concerned only negative emotions such as fear and sadness, which now would be interpreted as a lack of affective ToM, and not a deficit in cognitive ToM. Dysfunctions in ToM in persons with psychopathic traits are thus subtle and may be interpreted in a way that is not done so in previous studies.

### The Default Mode Network

Throughout the years, studies examining neuronal networks involved in psychopathic personality have increasingly been carried out, for example by using functional connectivity analysis. Functional connectivity is defined as the relation between the neuronal activation patterns of anatomically separated brain areas. Psychopathy has mostly been associated with atypical functional connectivity in (regions of) the default mode network (DMN; Raichle, 2015), including the mPFC, posterior cingulate cortex, precuneus, and angular gyrus, as well as bilateral IPL expanding to posterior temporal areas around the TPJ (Buckner et al., 2008). The DMN has been implicated in empathy, self-processing and moral behavior (Buckner et al., 2008; Andrews-Hanna et al., 2010; Li et al., 2014), and abnormal functioning of this network may play an important role in explaining core psychopathic traits, such as impaired emotion recognition (e.g., affective ToM; Grimm et al., 2009), and impaired moral decision making (Tassy et al., 2013). Subsequently, the DMN now is becoming increasingly recognized as a network of the social brain (Mars et al., 2012).

To sum up, given the above reviewed literature, we may conclude that individuals with psychopathic traits are found to have a deficit in dispositional empathy, particularly related to the processing of distress and negative arousal cues (i.e., affective empathy and affective ToM). These deficits are likely to be related to dysfunctions in a wide brain network involved in empathy, including the vmPFC/OFC and amygdala. And because a lack of sharing of vicarious negative arousal in these individuals, this may result in not showing empathic concern for others. In other words, individuals with higher levels of psychopathic traits show weaker psychophysiological reactions to these negative arousal cues and have poor aversive conditioning and stimulus-reinforcement learning. However, it is important to mention some limitations to the above conclusion. One is that other brain systems are also important in mediating other psychopathic personality traits, such as impulsivity and other impairments in executive functioning (see Koenigs et al., 2011 for a review). However, reviewing these traits is not within the scope of this review on the social brain.

Also important, studies reviewed in this review largely involved neuroimaging studies using fMRI. Within the social neuroscience of empathy in psychopathic personality, studies using electrophysiological measurements are scarcer. Electrophysiological studies are of additional value here, for example because it gives insight in the functional dynamics of different processes in higher temporal resolution compared to fMRI. Also, studies involving empathy mainly have focused on empathy for pain. For future research, it is very important to elucidate further the electrophysiological correlates of empathy in relation to psychopathic traits using ecologically more valid stimuli in tasks, such as pictures depicting aggressive situations (see for example van Dongen et al., 2018), but also other forms of empathy, for instance “positive empathy” (see Morelli et al., 2015). When doing so, this gives more insight in the social neuroscience aspects of empathy, not only the sensory aspects when the processing of pain stimuli is involved. Moreover, using aggression scenes or pictures depicting victims in distress is of particular importance, because of its ecological value when studying psychopathic personality.

## The Missing Link: the Way Forward

Research has mainly relied on social- and behavioral sciences when studying psychopathic personality. This makes sense, because psychopathic personality manifests itself most apparently at the surface with behavior that deviates from the social norm. Also, as with some forms of psychopathology, psychopathic personality has been generally viewed as a *mental* disorder. Though, as became clear in the current review, a shift from investigating forensic and correctional samples to community-based samples, accompanied by a shift from a diagnostic to dimensional perspective of psychopathic traits, has long been underway. Also, using classification based on overt behavior, we risk failing to identify important mechanisms involved in the psychopathology of psychopathic personality traits. For instance, assessments and tasks that are used to assess levels of empathy in this personality may not be sensitive enough to detect particular deficits in empathic abilities (Shamay-Tsoory et al., 2010; Domes et al., 2013). Thus, although the general view is that psychopaths lack affective empathy and have intact ToM, this may be challenged when using more sensitive ToM tasks. Moreover, when no overt behavioral differences between individuals scoring high and low on psychopathic traits are found, this may not automatically reflect “true” underlying resemblance in neurophysiological mechanisms. Also, when no behavioral differences are found, but underlying automatic (neural) processes differ in individuals with psychopathic traits, this may affect automatic responding outside the laboratory (e.g., Meffert et al., 2013). This points to the idea that, when necessary, psychopaths may use covert (computational) strategies in the brain to overcome otherwise automatic inappropriate responding.

In addition, as in this review discussed, complex and multifaceted nature of psychopathic personality, it is crucial to use additional neuroscientific insights to understand an individual (assessment) and for subsequent (targeted) effective treatment of higher levels of psychopathic personality. It has become clear that without neuroscience, the possibility to form a complete picture of psychopathologies and personalities, including psychopathic personality, is clearly missed. Hence, like mental disorders (Insel and Cuthbert, 2015), psychopathy now can be viewed as a disorder of the brain. Also, the influence of neuroscience in social science is not only important for a better understanding of the etiology, different expressions, and phenotypes of psychopathy, but also for the development of effective interventions. Because of the trial and error nature of interventions to date, much of these interventions are found not to be much effective (e.g., Salekin et al., 2010). By elucidating the underlying mechanisms that motivate persons with psychopathic traits in their behavior, interventions can be developed more targeted at specific dysfunctional mechanism, such as deficient dispositional empathy.

**FIGURE 1:**
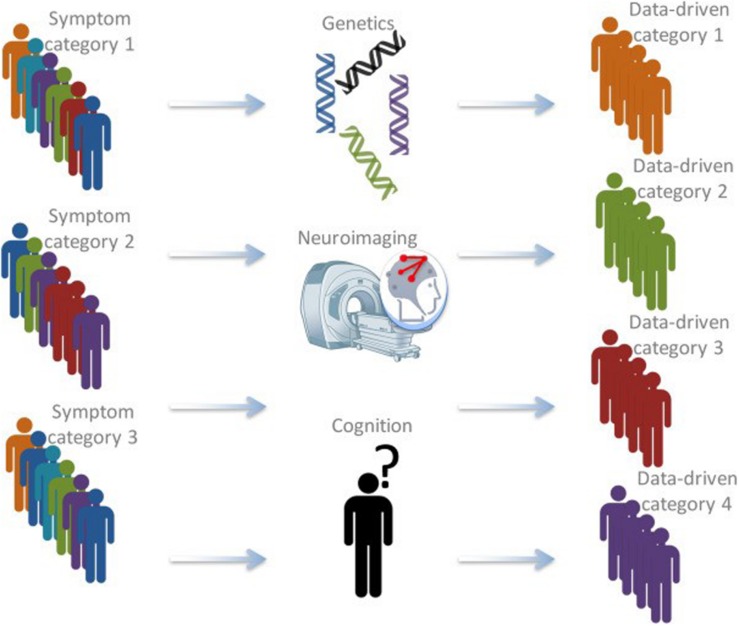
Schematic depiction of the bio-cognitive approach (after Insel and Cuthbert, 2015). Currently, patients are often categorized based on behavioral symptoms. Using information from a variety of approaches, including genetics, structural and functional neuroimaging, cognitive measures, and computational psychiatry, latent categories that might be much better at grouping different patients and predicting therapy outcomes might be found (Brazil et al., 2018).

During the last decade, insights from (neuro)biology with psychology and psychiatry are increasingly combined to form a new basis for categorizing individuals (see Figure 1). Most prominent is the approach that has been put forward in the Research Domain Criteria (RDoC) framework, developed by the National Institute of Mental Health (NIMH; Insel et al., 2010). This framework aims to understand mental illness as the interaction of factors at multiple levels (i.e., genetically, neurologically, behavioral, etc.). Most importantly, it calls for a stop in linking specific biological or cognitive factors to broad diagnostic (based on the DSM) disorders (Insel and Cuthbert, 2015).

**FIGURE 2:**
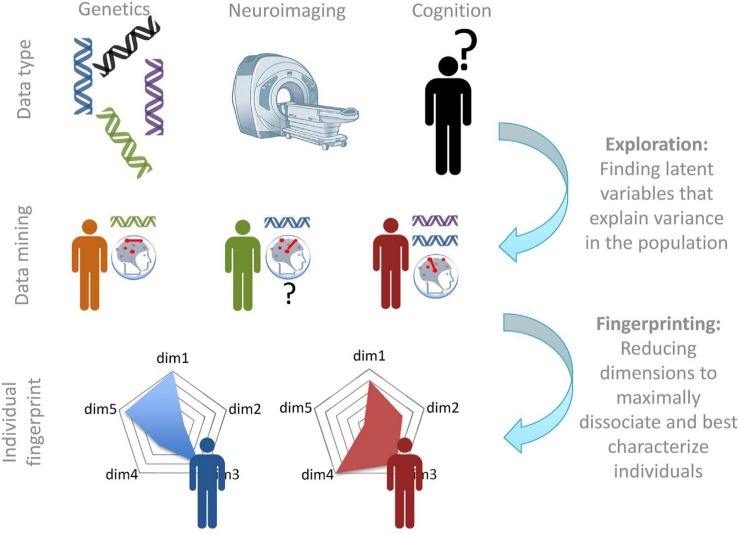
Exploration and fingerprinting of multimodal data. Once the required data are available, various data mining approaches will be required to determine new categories and the measures that describe them. In the second step, the most diagnostic measures can be summarized in a “fingerprint” or profile that can be used to describe each individual on a number of continuous latent dimensions (Brazil et al., 2018).

Recently, a converging framework has been put forward that applies such approach to antisocial individuals, including individuals with high levels of psychopathic traits (Brazil et al., 2018). In this bio-cognitive approach, it is suggested to use information from different levels, to form latent categories on which individuals are grouped, that may be better reflect underlying (neurobiological) dysfunctions. Subsequently, these newly defined latent categories may be more effective in guiding interventions and treatment. The approach will use different types of data (i.e., genetics, neuroimaging, cognition) to develop “fingerprints” of individuals that describe that individual based on their unique combination on different dimensions (see Figure 2).

Neurophysiology to guide personalized medicine has already been proved to be very promising in another domain of psychiatry, that of depression. Using data from a consortium, Drysdale et al. (2017) used fMRI connectivity analyses to form “biotypes” on the basis of dysfunctional connectivity patterns. These subtypes of depression were also related to effectiveness of transcranial magnetic stimulation. The authors also pointed to the importance of creating profiles of neurophysiological dysfunction that cross diagnostic boundaries and that can ultimately guide targeted intervention.

However, despite new insights in the complex nature of brain networks (as described in the previous section), there is a lack of studies investigating neural communication within specific frequency bands in psychiatry in general and psychopathic personality more specifically. Moreover, there is a lack of studies that look into dysfunctional topological properties of neural communication within these neural networks (Bullmore and Sporns, 2009). Previous studies are unable to directly evaluate how psychopathy-related connectivity abnormalities actually impact the efficiency and effectiveness of neural information transfer and integration. Also, given the complex structure of psychopathic personality, it is likely that particular traits within psychopathic personality (i.e., more related to F1 or F2 traits, or boldness, meanness, or disinhibition) are differentially associated with complex brain networks in different frequency bands, and with different topological properties of the functional connectivity. In a recent study, Tillem et al. (2018) applied a novel graph theory analysis, minimum spanning tree (MST) analysis, to resting-state EEG data. They found that the interpersonal-affective traits of psychopathy (F1) were associated with decreased efficiency in neural communication between both local and distal brain regions. Conversely, the impulsive-antisocial traits of psychopathy (F2) were associated with increased efficiency of neural communication between both local and distal brain regions.

In my view, the future of an understanding of empathy in psychopathic personality lies with studying the complex networks in the brain in combination with the use of other levels of information (i.e., genetics and cognition). Based on that, profiles of individuals can be formed that can be used to guide neurophysiological informed personalized treatment interventions that ultimately reduce violent transgressions in individuals with psychopathic traits. For example, using brain modulation techniques such as transcranial direct current stimulation (tDCS), activity in particular neural networks can be modulated, thereby modulating its activation and related cognition or behavior in treated individuals. For instance, a study by Choy et al. (2018) showed that when modulating activity with tDCS in the prefrontal cortex, healthy adult individuals were less intended to use aggression during an aggression task. These results point out that tDCS might be a promising alternative treatment for forensic populations (see for example Sergiou et al., 2020).

## Conclusion

In sum, in this review, the current knowledge on the social neuroscience of empathy in psychopathic personality is discussed, thereby contributing to a better insight in the empathic brain of psychopaths. It is argued that it is important to incorporate data from neuroscience in social sciences, because behavior, especially within the laboratory during experiments, will not reveal the whole picture behind this complex personality. Social neuroscience may unravel differences in functional brain networks that relate to the “empathic brain” of persons with elevated levels of psychopathic personality. Insight in these different complex relations will ultimately lead to a better understanding of this personality and how to target dysfunctional behavior accompanying this personality (e.g., aggression and violence).

To go forward, there is a need for a new approach in studying complex mechanisms, such as empathy, in psychopathic personality. I think that the new way forward must be based on frameworks (e.g., Insel et al., 2010; Brazil et al., 2018) that underscore the need of integration of multiple levels of data types, including neurobiological based information to classify psychopathic personality. By doing so, precision medicine (or personalized medicine; Wium-Andersen et al., 2017) will become a very promising new treatment strategy that can guide social science, including psychology, in developing new and effective interventions for psychopathy.

## Author Contributions

JD has developed the idea for the review and has written the whole manuscript.

## Conflict of Interest

The author declares that the research was conducted in the absence of any commercial or financial relationships that could be construed as a potential conflict of interest.
